# Motion Intent Recognition in Intelligent Lower Limb Prosthesis Using One-Dimensional Dual-Tree Complex Wavelet Transforms

**DOI:** 10.1155/2021/5631730

**Published:** 2021-11-24

**Authors:** Min Sheng, Wan-Jun Wang, Ting-Ting Tong, Yuan-Yuan Yang, Hui-Lin Chen, Ben-Yue Su

**Affiliations:** ^1^School of Mathematics and Physics, Anqing Normal University, Anqing 246133, Anhui, China; ^2^School of Mathematics and Computer, Tongling University, Tongling 244061, China; ^3^Key Laboratory of Intelligent Perception and Computing of Anhui Province, Anqing Normal University, Anqing 246133, China

## Abstract

The motion intent recognition via lower limb prosthesis can be regarded as a kind of short-term action recognition, where the major issue is to explore the gait instantaneous conversion (known as transitional pattern) between each two adjacent different steady states of gait mode. Traditional intent recognition methods usually employ a set of statistical features to classify the transitional patterns. However, the statistical features of the short-term signals via the instantaneous conversion are empirically unstable, which may degrade the classification accuracy. Bearing this in mind, we introduce the one-dimensional dual-tree complex wavelet transform (1D-DTCWT) to address the motion intent recognition via lower limb prosthesis. On the one hand, the local analysis ability of the wavelet transform can amplify the instantaneous variation characteristics of gait information, making the extracted features of instantaneous pattern between two adjacent different steady states more stable. On the other hand, the translation invariance and direction selectivity of 1D-DTCWT can help to explore the continuous features of patterns, which better reflects the inherent continuity of human lower limb movements. In the experiments, we have recruited ten able-bodied subjects and one amputee subject and collected data by performing five steady states and eight transitional states. The experimental results show that the recognition accuracy of the able-bodied subjects has reached 98.91%, 98.92%, and 97.27% for the steady states, transitional states, and total motion states, respectively. Furthermore, the accuracy of the amputee has reached 100%, 91.16%, and 90.27% for the steady states, transitional states, and total motion states, respectively. The above evidence finally indicates that the proposed method can better explore the gait instantaneous conversion (better expressed as motion intent) between each two adjacent different steady states compared with the state-of-the-art.

## 1. Introduction

The 2011 World Disability Report points out that there are at least 30 million amputees in developing countries [1–4]. The prosthesis can allow amputees to maintain the limb balance and compensate for the body appearance. It also improves the amputees' integration into society and restores their ability to works [5]. Therefore, the prosthesis designers attempt to use engineering methods to design a variety of prostheses that meet the needs of amputees.

The motion intent recognition via the lower limb prosthesis requires identifying the gait instantaneous conversion (known as transitional pattern) between each two adjacent different steady states of gait mode. Current research studies on this issue mainly rely on the human surface electromyographic signals (sEMGs) [6] or mechanical signals [7]. The sEMGs [8], which are collected by the biological electrode attached to the skin surface, can reflect the muscle contraction and relaxation. Due to the characteristics, the motion intent recognition based on sEMGs has been widely studied during these years. For example, Huang et al. [9] proposed a sEMGs-based human motion recognition method, which successfully recognized 7 daily motion states. By calculating a set of statistical features (average value and standard deviation) and using the linear discriminate analysis (LDA), it is reported that the average recognition accuracy has reached 92.6% [9]. Note that, although the sEMGs can better reflect the strength of muscle contraction, they are easily affected by the nerve atrophy and electrode position [10].

The signals collected by mechanical sensors can avoid the above drawbacks of sEMGs, so they are widely used for the motion intent recognition in recent years. The mechanical signals [11–13], which are collected by the accelerometers, gyroscopes, pressure sensors, or other devices, can reflect the kinematics and power information (e.g., acceleration, angular velocity, joint angle, and ground contact force). In the study of motion intent recognition based on mechanical signals, Liu et al. [14] defined 15 motion states based on 5 steady states with three different speed levels. In this method, by using the data fusion theory, the intraclass correlation coefficient (ICC) of the data is calculated. Then, the motion states are recognized by the hidden Markov model (HMM), which results in an accuracy of 95.8%.

In this paper, we propose an improved motion intent recognition method based on the mechanical signals via intelligent lower limb prosthesis. The main contributions of this paper are listed as follows:

1. We introduce the one-dimensional dual-tree complex wavelet transform (1D-DTCWT) to study the transitional pattern between two adjacent different steady states so as to identify the motion intent of the lower limb amputees. The wavelet transform has the ability of the time-frequency local analysis, which can amplify the instantaneous variation characteristics of gait information, making the extracted features of instantaneous pattern more stable.
2. Because of the continuity of human lower limb movement, it is meaningful to explore the continuity features of the discrete signal before classifying the amputee's motion intent. Fortunately, the translation invariance and direction selectivity of 1D-DTCWT can help to explore the continuous features of patterns. The extracted features thereby can better reflect the inherent continuity of human lower limb movements, which further improves the recognition accuracy.
3. The method in this paper has been compared with some proposals in the related literature, and most existing intent recognition methods usually adopt a set of statistical features to classify the motion patterns. We have used 1D-DTCWT to select the inherent continuity features of motion for intent recognition and achieving good recognition performance.

## 2. Related Works

### 2.1. Mechanical Signal-Based Motion Intent Recognition

There are a variety of mechanical signals-based methods for the motion intent recognition of the unilateral lower limb amputee. Traditional intention recognition methods typically follow such a framework. They firstly collect the sensor data from the affected side and then extract a set of statistical features from the processed data for the pattern recognition. For example, Zheng et al. [15] combined the pressure sensor and inertial measurement unit to collect the affected side data, extracted the mean, maximum, and standard deviation as features, and used SVM classifier to recognize the six steady-state patterns, with a recognition rate of 92.7%. However, this method does not involve the identification of transitional states. Young et al. [16] collected the data by using an inertial measurement unit and a pressure sensor from eight amputees' affected sides. By selecting statistical features (mean and variance values) and using a deep belief network (DBN) classifier, the recognition accuracy of 13 motion states has reached about 90%. Note that, the above method uses the collect data from the affected side, which will result in the lag of motion intent.

With the rapid development of wearable technology, wearable devices [17, 18] are becoming smaller and more convenient to carry, which is conducive to placing sensors on the healthy side to collect data. Su et al. considered that mapping the healthy side motion data collected by the sensor to the prosthesis control system makes the prosthesis to predict the motion changes in advance, so that the movement of the amputee can be more stable and smoother by improving the corresponding control strategy. Therefore, Su et al. proposed methods to place the sensors on the healthy side for recognizing the motion intent of the unilateral lower limb amputee [19–21].

Su et al. [19] redefined the motion pattern of intelligent lower limb prosthesis, proposed to place the sensor on the healthy side to collect data, and used statistical features (mean, variance, maximum, and minimum) and SVM [22–26] classifier to perform feature extraction and classification. The recognition accuracy of 13 motion states reaches 95.12% (ten able-bodied subjects). Subsequently, Su et al. [20] used the Gaussian mixture model-hidden Markov model (GMM-HMM) to recognize the motion intent based on the angular velocity and acceleration signals obtained by the inertial sensor. The recognition accuracy of 13 motion states reaches 96.92% (ten able-bodied subjects). To avoid artificial selection of features, Su et al. [21] used a convolutional neural network (CNN) to self-select features for recognizing the motion intent of lower limb amputee. The recognition accuracy of 13 motion states reached 94.15%±3.04% (ten able-bodied subjects) and 89.23%±4.21% (one amputee subject). The above methods do not consider the continuity of human lower limb movement; it is important to study the continuity features of the discrete signal before classifying the motion intent.

### 2.2. Motivation

Most intent recognition methods usually employ a set of statistical features to classify the transitional patterns. However, the statistical features of the short-term signals via the instantaneous conversion are empirically unstable, which may degrade the classification accuracy. The reduction of recognition accuracy may increase the probability of amputee wrestling. Therefore, it is necessary to select stable motion features to improve the recognition accuracy of motion intention.

The key problem of lower limb prosthesis movement intent recognition is to study the gait instantaneous conversion (known as transitional pattern) between each two adjacent different steady states of gait mode. Wavelet transform can analyze the localization of time (space) frequency and gradually refine the signal through expansion and translation operation, so as to finally achieve time subdivision at high frequency and frequency subdivision at low frequency. It can automatically adapt to the requirements of time-frequency signal analysis, so as to focus on any detail of the motion signal. The local analysis ability of the wavelet transform can study the instantaneous variation characteristics of gait information, making the extracted features of instantaneous pattern between two adjacent different steady states more stable.

Considering the continuity of human lower limb motion, it is necessary to functionalize discrete motion data to obtain the continuous features of human motion. Firstly, 1D-DTCWT can provide details in six different directions, which is conducive to the study of transitional modes. Secondly, the half sampling delay between filters in 1D-DTCWT can make it approximately translation invariant and effectively suppress the aliasing of motion data, so as to explore the continuity characteristics of motion. Therefore, the translation invariance and direction selectivity of 1D-DTCWT can help to explore the continuous features of patterns, which better reflects the inherent continuity of human lower limb movements and further improves the recognition accuracy. Based on the above considerations, we introduce the 1D-DTCWT to solve the motion intent recognition via lower limb prosthesis.

## 3. Materials and Methods

In this section, we introduce the principles of 1D-DTCWT in detail, relevant definitions about the motions, the proposed motion intent recognition methods, data source and processing, and experimental classification strategy.

### 3.1. 1D-DTCWT Method

In 1998, Kingsbury [27] proposed DTCWT to perform complex wavelet transforms using real wavelet transforms to solve the problem that complex wavelet transform cannot be completely reconstructed. It not only offers the benefits of traditional wavelet transforms but also maintains support for multiple resolutions and time-frequency localized analysis [28–30].

The basic principle of 1D-DTCWT is derived from the Fourier transform. The wavelet function of 1D-DTCWT is shown in equations (1) and (2). The two real wavelet functions are used as real and imaginary parts of a plural form expression. The real and imaginary parts are approximate Hilbert transforms of each other.

(1) $$\begin{array}{c} \Psi \left(t\right)=\Psi_{h}\left(t\right)+j\Psi_{g}\left(t\right), \end{array}$$

(2) $$\begin{array}{c} \Psi_{g}\left(t\right)\approx H\left\{\Psi_{h}\left(t\right)\right\}. \end{array}$$

In equation (2), *H*{·} is the Hilbert transform operator.

**Figure 1:**
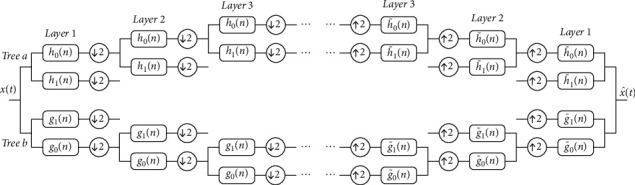
1D-DTCWT decomposition and reconstruction, where *h*_0_(*n*), *h*_1_(*n*), *g*_0_(*n*), *g*_1_(*n*), ${\tilde{h}}_{0}\left(n\right)$, ${\tilde{h}}_{1}\left(n\right)$, ${\tilde{g}}_{0}\left(n\right)$, and ${\tilde{g}}_{1}\left(n\right)$ denote filters, ↓2 indicates interleaved sampling, and ↑2 indicates interpolation: (a) decomposition process; (b) reconstruction process.

1D-DTCWT uses two parallel discrete wavelet trees, tree *a* and tree *b* (see Figure 1), independently to generate transformed real and imaginary parts. There is a delay between the trees, so the data obtained by real and imaginary transforms complement each other. Because tree *a* and tree *b* are transformed in parallel, the calculation processes for the trees are also independent of each other during decomposition and reconstruction. Tree *a* wavelet and scale coefficients are as shown in the following equations:

(3) $$\begin{array}{c} d_{j}^{Re}\left(n\right)=2^{j/2}\int_{-\infty }^{+\infty }\left(t\right)\Psi_{h}\left(2^{j}t-n\right)\text{d}t,\;\left(j=1,2,\ldots ,J\right), \end{array}$$

(4) $$\begin{array}{c} a_{J}^{Re}\left(n\right)=2^{J/2}\int_{-\infty }^{+\infty }x\left(t\right)\Psi_{h}\left(2^{J}t-n\right)\text{d}t, \end{array}$$

where *J* is the total number of decomposition layers. Similarly, the wavelet and scale coefficients for tree *b* are shown in the following equations:

(5) $$\begin{array}{c} d_{j}^{Im}\left(n\right)=2^{j/2}\int_{-\infty }^{+\infty }x\left(t\right)\Psi_{g}\left(2^{j}t-n\right)\text{dt},\;\left(j=1,2,\ldots ,J\right), \end{array}$$

(6) $$\begin{array}{c} a_{J}^{Im}\left(n\right)=2^{J/2}\int_{-\infty }^{+\infty }x\left(t\right)\Psi_{g}\left(2^{J}t-n\right)\text{d}t. \end{array}$$

Finally, the reconstruction expressions of 1D-DTCWT are shown in the following equations:

(7) $$\begin{array}{c} d_{j}\left(t\right)=2^{\left(j-1\right)/2}\left[\sum_{n=-\infty }^{\infty }d_{j}^{Re}\left(n\right)\Psi_{h}\left(2^{j}t-n\right)+i\sum_{n=-\infty }^{\infty }d_{j}^{Im}\left(n\right)\Psi_{g}\left(2^{j}t-n\right)\right], \end{array}$$

(8) $$\begin{array}{c} a_{J}\left(t\right)=2^{\left(J-1\right)/2}\left[\sum_{n=-\infty }^{\infty }a_{J}^{Re}\left(n\right)\Psi_{h}\left(2^{J}t-n\right)+i\sum_{n=-\infty }^{\infty }a_{J}^{Im}\left(n\right)\Psi_{g}\left(2^{J}t-n\right)\right], \end{array}$$

(9) $$\begin{array}{c} \tilde{x}\left(t\right)=\sum_{j=1}^{J}d_{j}\left(t\right)+a_{J}\left(t\right). \end{array}$$

Equation (9) can be expressed as the plural form as follows:

(10) $$\begin{array}{c} \tilde{x}\left(t\right)={\tilde{x}}^{Re}\left(t\right)+i{\tilde{x}}^{Im}\left(t\right). \end{array}$$

The signal amplitude envelope of 1D-DTCWT is

(11) $$\begin{array}{c} \hat{x}\left(t\right)=\sqrt{{\tilde{x}}^{Re}{\left(t\right)}^{2}+{\tilde{x}}^{Im}{\left(t\right)}^{2}}. \end{array}$$

In short, 1D-DTCWT consists of a cluster of wavelet basis functions which can analyze signals in any time or space domain. We adopt the 1D-DTCWT method to fit collected discrete motion data into a continuous curve and apply its local analysis capabilities to enlarge the instantaneous variation characteristics of gait information. 1D-DTCWT uses two-way complex wavelet transform with a binary tree structure to support translation invariance and direction selection, so it captures the continuous features hidden in the data that better reflect the continuity of human lower limb movement.

### 3.2. Human Lower Limb Motion States

Human lower limb motion has continuity and periodicity. According to the different roles played by the lower limb, the gait cycle is divided into the stance phase and the swing phase.

- Gait cycle: the gait cycle in horizontal ground conditions begins with the initial contact of the heel of one foot landing on the ground and ends when the same heel next lands on the ground
- Swing phase: within a gait cycle, the swing phase is the time required to move the nonweight-bearing foot forward, from the time the toe leaves the ground until the same heel lands again
- Stance phase: within a gait cycle, the stance phase is the time when the foot from the swing phase bears weight, reckoned from the time the heel lands on the ground until the toe lifts again
- Steady state: steady state describes motion taking place over constant terrain conditions
- Transitional state: it is the transitional state from an initial motion mode to another motion mode under different terrain conditions
- Transitional step: it starts from the toe off time of one foot in the previous terrain and ends at the heel landing of the same side foot in the latter terrain

From the perspective of pattern recognition, in order to facilitate the performance analysis of the test algorithm, our work requires distinguishing three types of motion.

The first type comprises the 5 steady states: level walking (LW), stair ascent (SA), stair descent (SD), ramp ascent (RA), and ramp descent (RD), as shown in Table 1.

The second type comprises the 8 transitional states: level walking to stair ascent (LW-SA), level walking to stair descent (LW-SD), level walking to ramp ascent (LW-RA), level walking to ramp descent (LW-RD), stair ascent to level walking (SA-LW), stair descent to level walking (SD-LW), ramp ascent to level walking (RA-LW), and ramp descent to level walking (RD-LW), as shown in Table 2.

The third type comprises the 13 total motion states combining the steady and transitional states. The 5 steady states are the most basic human lower limb motion states in daily life. The 8 transitional states reflect changes in terrain encountered in daily life.

### 3.3. Motion Intent Recognition Methods

**Figure 2:**
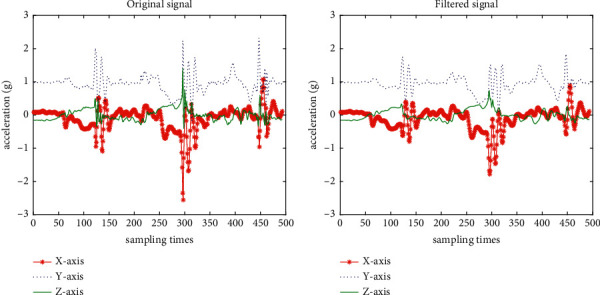
Acceleration signals of LW-SA (sensor data at the thigh): (a) original signal; (b) filtered signal.

Through experimental test and comparison, we use the moving average filter with the window width of 5 frames (sampling frequency of 96 Hz) to process the motion data (as shown in Figure 2).

**Figure 3:**
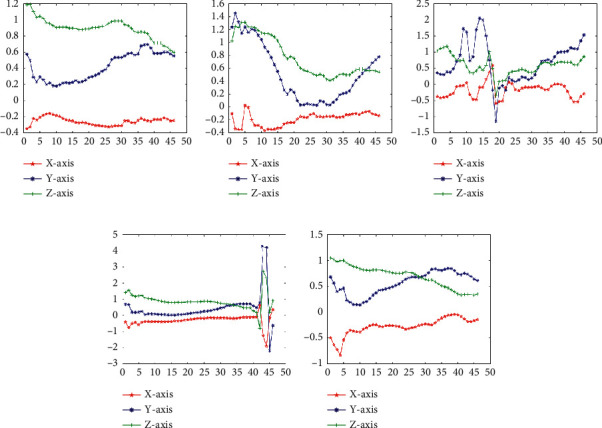
Acceleration signal fitting in steady states (sensor data at the thigh), where the ordinate represents acceleration (g) and the abscissa represents number of frames: (a) LW; (b) SA; (c) SD; (d) RA; (e) RD.

**Figure 4:**
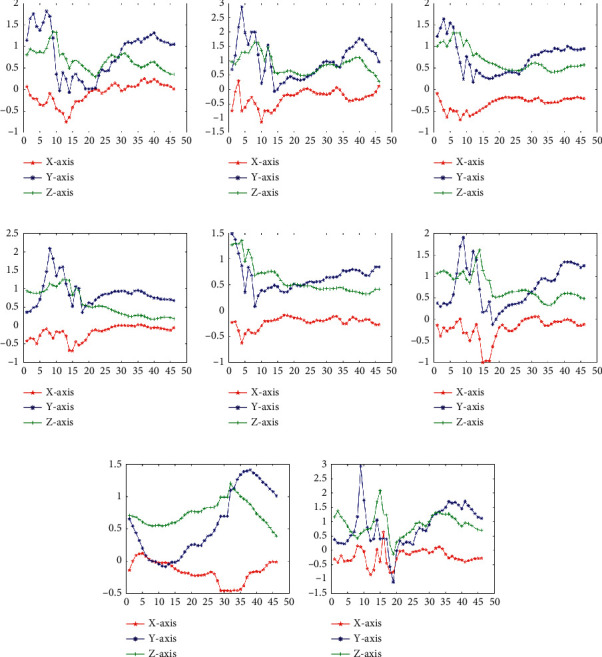
Acceleration signal fitting in transitional states (sensor data at the thigh), where the ordinate represents acceleration (g) and the abscissa represents number of frames: (a) LW-SA; (b) LW-SD; (c) LW-RA; (d) LW-RD; (e) SA-LW; (f) SD-LW; (g) RA-LW; (h) RD-LW.

Firstly, we through 1D-DTCWT to fitting processed discrete motion data into continuous curve (shown in Figures 3 and 4). Observing the blue fitting curve in Figure 3, it can be seen that the fitting curves of different motion states have different shapes, so using 1D-DTCWT can be divided into different motion states. The 1D-DTCWT has local analysis capabilities that can amplify the local information of motion so that it can better study the instantaneous variation characteristics of gait information.

**Figure 5:**
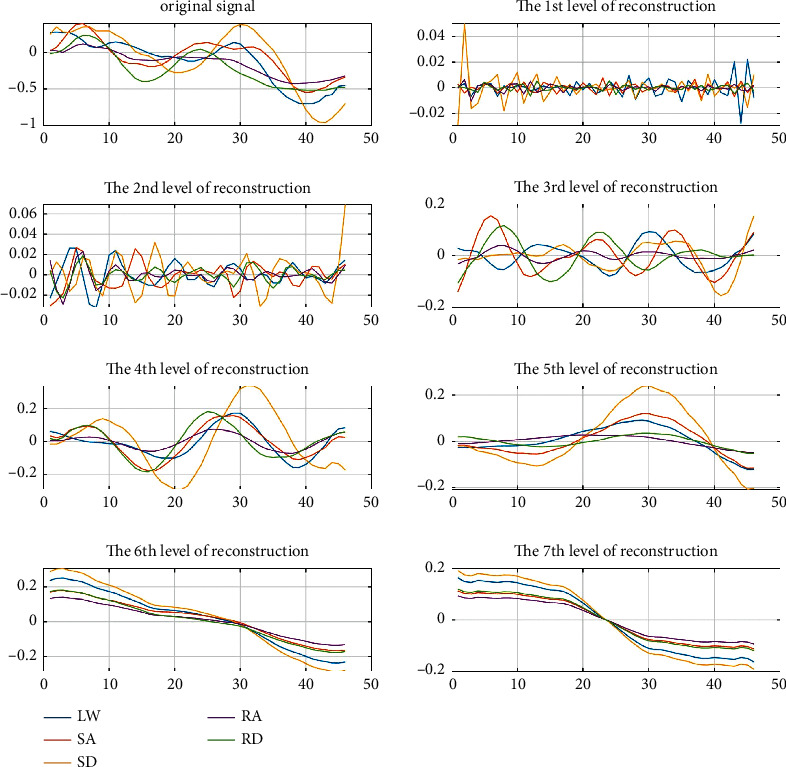
1D-DTCWT reconstruction of steady states (sensor data at the thigh), where the ordinate represents acceleration (g) and the abscissa represents number of frames.

**Figure 6:**
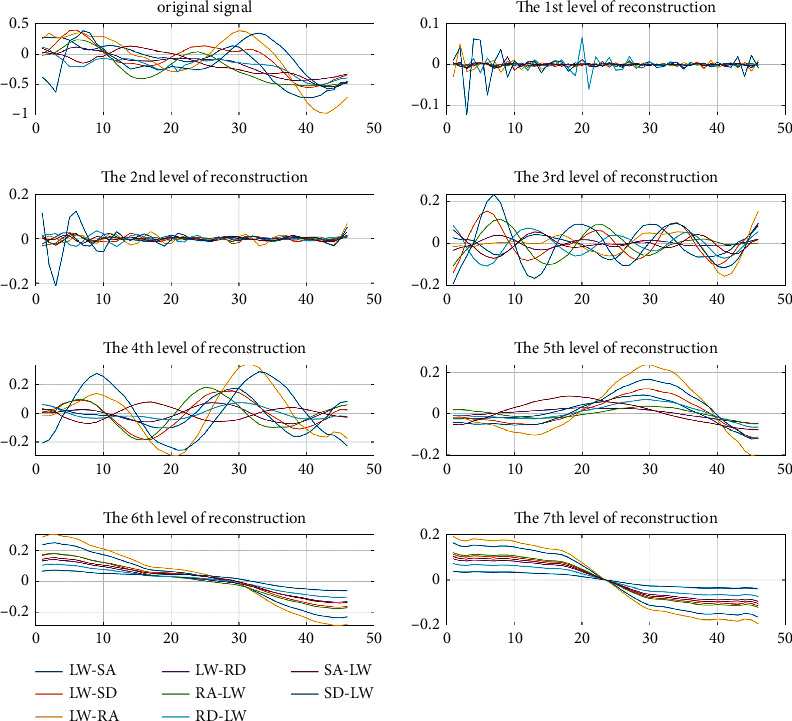
1D-DTCWT reconstruction of transitional states (sensor data at the thigh), where the ordinate represents acceleration (g) and the abscissa represents number of frames.

Secondly, the analysis of the reconstruction of different layers of 1D-DTCWT revealed that the five-layer reconstruction makes the curve fitting difference of motion modes most obvious shown in Figures 5 and 6, where the ordinate represents acceleration and the abscissa represents the time interval. Therefore, we selected these *i*^*th*^-layer{*i*=5 in this paper) of low-frequency coefficients as the continuous features of human lower limb motion that best retains the continuous information of the motion.

**Figure 7:**
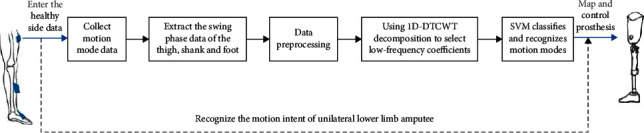
Motion intent recognition process.

At last, SVM is selected to classify and recognize 72 (3 ×6× 4, where 3 represents three sensors, 6 is composed of triaxial acceleration and triaxial angular velocity, and 4 represents the number of five-layer low-frequency coefficients of 1D-DTCWT) dimensional feature vector. The proposed overall motion intent recognition process is shown in Figure 7. The general framework of the proposed motion intent recognition method is shown in Algorithm 1.

### 3.4. Data Source and Processing

This article uses the data set of literature [21]. The experiments recruited ten able-bodied subjects and one transfemoral subject. The ten able-bodied subjects (five males and five females), varied in age (18–30), height (1.58–1.83 m), and weight (40–86 kg). The transfemoral subject was 67 years old and had been wearing his Teh Lin-model prosthesis for 12 years. The experimental environment includes a staircase with a step height of 16 cm and a ramp with a slope of 10°. All experimental data were collected under physician's guidance. The experimenters are responsible for collecting and recording the time series data generated by the sensor. In the actual test, lower limb amputees will automatically adjust the step sequence when performing transitional states. The step sequence of experimental data collection is shown in literature [21].

Three inertial sensors from Noitom Perception Legacy are placed on the thigh, shank, and foot of subject healthy. Each inertial sensor consists of a three-axis accelerometer and a three-axis gyroscope, and the sampling frequency is 96 Hz. For the five steady states, we determined the starting point of the toes leaving the ground according to the ground contact state sequence obtained by the inertial sensor and then extracted the data corresponding to the transitional state. For the eight transitional states, the ground contact state obtained by the inertial sensor was used to find the starting point of the transitional step, and the window data were extracted from the starting point of the transitional step. The extracted window length data come from the swing phase. 1D-DTCWT uses two-way complex wavelet transform with a binary tree structure. Therefore, the frames of the window length are even number. After experimental test and comparison, the optimal number of frames is set to 46 frames in this paper.

### 3.5. Classification Types

In order to verify the robustness and effectiveness of the algorithm, this paper adopts user-independence and user-dependence strategies (Table 3). *K*-fold cross validation is used to evaluate the prediction performance of the model; 10-fold cross validation is a commonly-used method in action recognition field [31]. We conducted user-dependent classification tests on the ten able-bodied subjects and one amputee subject. For the ten able-bodied subjects, we used 10-fold cross validation to evaluate the performance of the algorithm. Specifically, the data sets were divided into 10 equal-sized subsets. The experiment used the union of nine subsets as the training set and the remaining subset as the test set. For the amputee subject, 90% of the samples were randomly selected as training data, and the remainder were selected as test data. To test the independence of the algorithm, we performed a user-independent classification test on the ten able-bodied subjects. In the user-independence test, we randomly selected samples from nine able-bodied subjects as the training set and the remaining subject's data as the test set.

## 4. Experiments and Results

### 4.1. Experimental Results

#### 4.1.1. User-Dependent Classification

**Figure 8:**
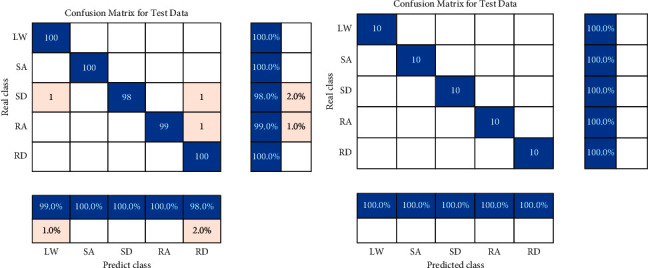
Confusion matrix of steady states for user-dependent classification: (a) able-bodied subjects; (b) amputee subject.

Figure 8 shows the confusion matrix of steady states for user-dependent classification. The confusion matrix describes the classification accuracy of each motion state, with the correctly classified test samples located on the diagonal. For the able-bodied subjects, the testing accuracy reached 98.91%±0.19% in steady states.  Figure 8(a) shows that stair descent (SD) had a slightly lower recognition than the other four steady states. Steady states such as stair descent (SD) were sometimes unrecognized as ramp descent (RD) due to the high degree of similarity between the movements in steady states. For the amputee subject, the testing accuracy reached 100%±0.00%. The result may be related to the length of time the amputee subject wears the prosthesis. The steady states are such common movements in daily life, and it is very important for amputees to more accurately recognize them.

**Figure 9:**
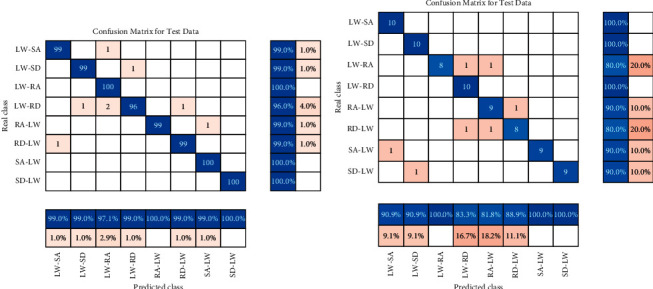
Confusion matrix of transitional states for user-dependent classification: (a) able-bodied subjects; (b) amputee subject.

Figure 9 shows the confusion matrix of transitional states for user-dependent classification. For the able-bodied subjects, the testing accuracy reached 98.92%±0.12% in transitional states.  Figure 9(a) shows that the recognition accuracy of level walking to ramp descent (LW-RD) was slightly lower than that of the other transitional states. Level walking to ramp descent (LW-RD) was sometimes mistakenly recognized as level walking to stair descent (LW-SD) possibly because the movement postures of ramp descent (RD) and stair descent (SD) are similar and difficult to distinguish. For the amputee subject, the testing accuracy reached 91.16%±1.28%. The decrease in results was likely related to the adjustment of the stepping order of the amputee when making transitional steps.

**Figure 10:**
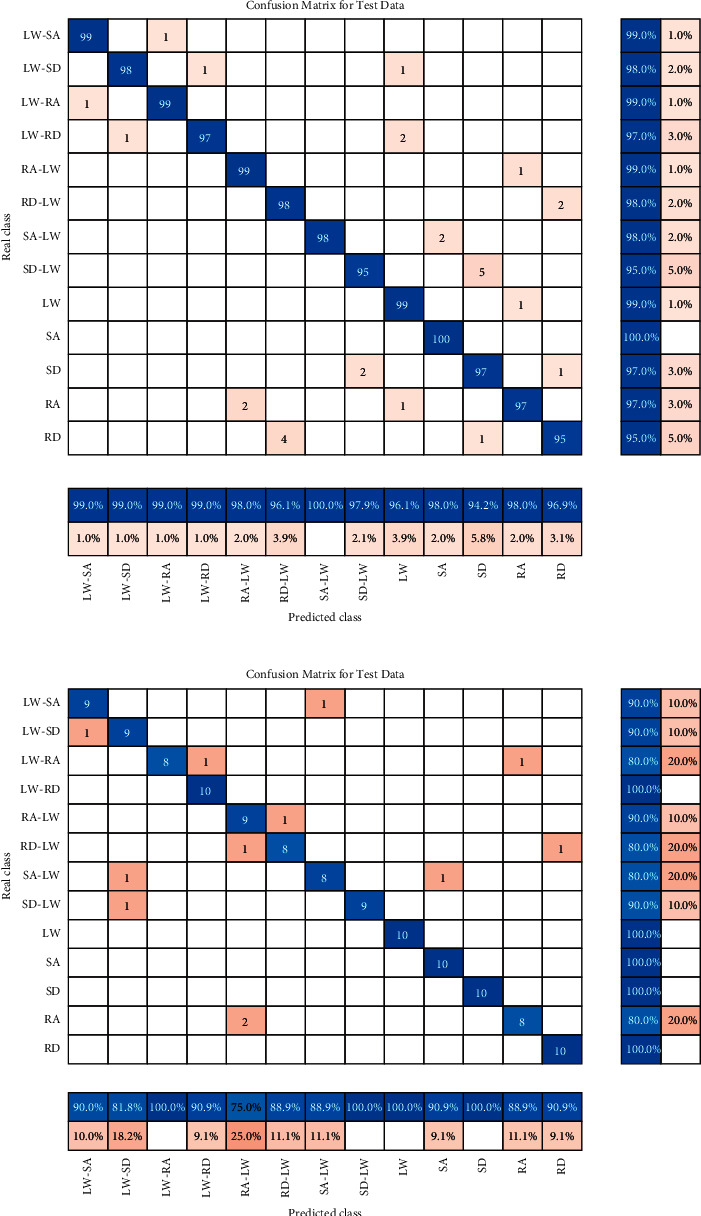
Confusion matrix of motion states for user-dependent classification: (a) able-bodied subjects; (b) amputee subject.

Figure 10 shows the confusion matrix of motion states for user-dependent classification. For the able-bodied subjects, the testing accuracy reached 97.27%±0.14% in motion states.  Figure 10(a) shows that the recognition rate of stair descent to level walking (SD-LW) was slightly lower than that of the other motion states. The transitional state of stair descent to level walking (SD-LW) was sometimes mistakenly recognized as the steady state of stair descent (SD). This may be due to the inertia of stair descent to level walking (SD-LW) that results in similar postures between stair descent (SD) and stair descent to level walking (SD-LW). For the amputee subject, the recognition accuracy reached 90.27%±1.23%. Since data collection involves privacy protection, ethical review, and so on, data on amputees are more difficult to obtain. This reduction in recognition accuracy may be related to the smaller amount of data for amputees. As the motion states increase, it may lead to insufficient classifier training, thereby affecting the recognition accuracy.

#### 4.1.2. User-Independent Classification

**Figure 11:**
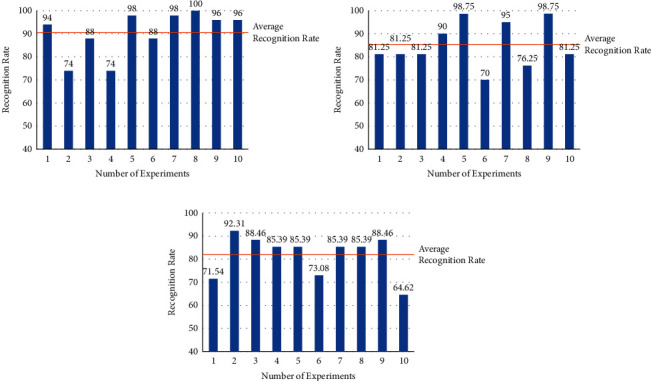
Tree diagram of able-bodied subjects' user-independent classification: (a) steady states; (b) transitional states; (c) motion states.

As mentioned above, data on amputation subjects are difficult to obtain, so independent user experiments were conducted on ten able-bodied subjects. The recognition results for steady states, transitional states, and motion states were 90.60%±5.96%, 82.01%±5.56%, and 82.01%±5.56%, respectively.  Figure 11 shows the tree diagram of part of the experimental results for the able-bodied subjects' user-dependent classification. The recognition results of most subjects fluctuate around the average value of recognition accuracy. Due to the individual differences of subjects, the recognition rate of individual subjects may have been slightly lower.

### 4.2. Analysis of Results

Table 4 shows how our method compares with other research methods for motion intent recognition. From the position of the sensor, literatures [14, 16, 32] collect data on the affected side. The methods in this paper and literatures [19, 21] use the same identification strategy; that is, the sensors are placed on the healthy side to collect data. When the movement of the affected side does not occur, the movement of the healthy side is identified and mapped to the affected side to identify the movement intention of the amputee. Therefore, the movement intention of the amputee can effectively avoid the lag problem, and the recognition rate is slightly higher than that of literatures [19, 21].

From the perspective of feature extraction, literatures [14, 16, 19, 32] use statistical features (e.g., mean, standard deviation, and variance) and literature [21] uses CNN self-selection features. Most intention recognition method selects a set of statistical features from discrete motion data to classifying motion patterns. However, the statistical characteristics of short-term samples are empirically unstable, which may reduce the classification accuracy. The self-selection feature of CNN can not accurately reflect the continuity of lower limb movement. Consider that the key of motion intent recognition is to study the transitional pattern between each two adjacent different steady states of gait mode. Therefore, it is necessary to use 1D-DTCWT to functionalize the discrete motion behavior data and use its local analysis ability, the translation invariance, and direction selectivity to explore the continuity features of transitional state stability. The experimental results finally indicate that the proposed method can better explore the gait instantaneous conversion (better expressed as motion intent) between each two adjacent different steady states compared with the state-of-the-art.

In terms of recognition accuracy, the recognition accuracy of 5 steady states, 8 transitional states, and 13 motion states in this method is higher than that in other literatures. The improvement of recognition accuracy can reduce the probability of wrestling and make the amputee's movement more smooth and stable.

Table 5 shows the comparison of different methods using the same data set. Taking into account the differences in gait between the able-bodied and amputee subjects, we performed this experiment for the subjects separately. Due to the inherent continuity of human lower limb movement, the use of statistical learning methods to extract a set of statistical features and CNN method self-selection features cannot more accurately reflect the continuity of lower limb motion. This paper uses the translation invariance and direction selectivity of 1D-DTCWT to explore continuous features closer to the nature of motion.

## 5. Conclusions and Discussion

In this paper, we propose a method of motion intent recognition using 1D-DTCWT, which is able to explore continuous features of stability for use in intelligent lower limb prosthesis. The proposed method is demonstrated to be able to solve the problem of the unstable statistical features of the short-term signals obtained by instantaneous conversion and to better study the gait instantaneous conversion between each two adjacent different steady states of gait mode. Moreover, continuous features excavated by 1D-DTCWT are more in line with the continuity of lower limb motion. The experimental results based on user-dependent and user-independent classification strategy have demonstrated the effectiveness and feasibility of the proposed method.

Although this research provides an alternative method for motion intent recognition in lower limb prosthesis, there are still some limitations. Firstly, future research should collect data on more amputees to improve the application of motion intent recognition field. Secondly, 1D-DTCWT will be integrated into the deep learning framework as a kernel function to self-select appropriate features to recognize the motion intent of the lower limb amputee. Finally, the effect of stride length on the motion intent recognition in lower limb amputees should be considered.

## Figures and Tables

**Algorithm 1:**
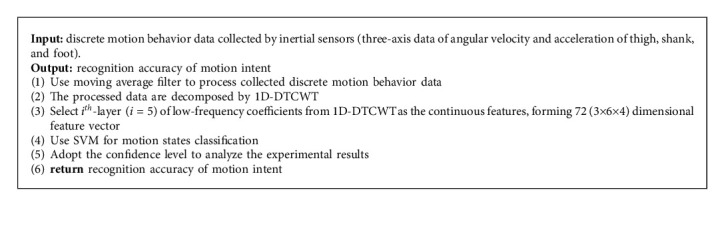
The proposed motion intent recognition.

**Table 1:** Steady states.

Number	Abbreviation	Details
1	LW	Level walking
2	SA	Stair ascent
3	SD	Stair descent
4	RA	Ramp ascent
5	RD	Ramp descent

**Table 2:** Transitional states.

Number	Abbreviation	Details
1	LW-SA	Level walking to stair ascent
2	LW-SD	Level walking to stair descent
3	LW-RA	Level walking to ramp ascent
4	LW-RD	Level walking to ramp descent
5	SA-LW	Stair ascent to level walking
6	SD-LW	Stair descent to level walking
7	RA-LW	Ramp ascent to level walking
8	RD-LW	Ramp descent to level walking

**Table 3:** User-independent and user-dependent classification strategies.

Classification type	Subjects	Training data	Testing data	Cross validation
User-dependent	Ten able-bodied subjects	90% of ten able-bodied subjects sample	10% of ten able-bodied subjects sample	Across subjects (×10)
	One transfemoral subject	90% of subject sample	10% of subject sample	Within subject (×10)
User-independent	Ten able-bodied subjects	Samples from nine subjects	Samples from one subject	Across subjects (×10)

**Table 4:** Comparison of our method with other methods under user-dependent classification.

Reference	Subjects	Position of sensors	Feature extraction	Classifier	Type of motion state		Accuracy
					Steady	Transitional	
Young et al. 2016 [16]	Eight transfemoral amputees	Prosthesis	Statistical features	DBN	5	8	90.00%
Liu et al. 2017 [14]	Three able-bodied two amputees	Prosthesis	Statistical features	HMM	5	\	95.80%
Zheng et al. 2017 [32]	Six transfemoral amputees	Prosthesis	Statistical features	SVM + QDA	\	8	94.90%
Su et al. 2020 [19]	Ten able-bodied	Healthy side	Statistical features	SVM	5	8	95.12%
Su et al. 2019 [21]	Ten able-bodied	Healthy side	Self-selected features from CNN	Softmax	5	8	94.15%±3.04%
	One amputee						89.23%±4.21%
Our method	Ten able-bodied	Healthy side	Five-layer low-frequency coefficients of 1D-DTCWT	SVM	**5**	—	98.91%±0.19%
					—	**8**	98.92%±0.12%
					**5**	**8**	97.27%±0.14%
	One amputee				**5**	—	100%±0.00%
					—	**8**	91.16%±1.28%
					**5**	**8**	90.27%±1.23%

**Table 5:** Comparison of our method with other methods with the same data set under user-dependent classification.

Subject	Position of sensors	Feature extraction	Classifier	Type of motion state		Accuracy
				Steady	Transitional	
Ten able-bodied	Healthy side	Mean, variance, and so on	SVM	5	8	95.12%
Ten able-bodied	Healthy side	—	GMM-HMM	5	8	96.92%
Ten able-bodied	Healthy side	Self-selection feature of CNN	Softmax	5	8	94.15%±3.04%
One amputee						89.23%±4.21%
Ten able-bodied	Healthy side	Five-layer low-frequency coefficients of 1D-DTCWT	SVM	5	—	98.91%±0.19%
				—	**8**	98.92%±0.12%
				**5**	**8**	97.27%±0.14%
One amputee				**5**	—	100%±0.00%
				—	**8**	91.16%±1.28%
				**5**	**8**	90.27%±1.23%

## Data Availability

Since data collection involves ethical review and privacy protection of volunteers, the data set will not be made public for the time being. The data used for the production of the results of the paper available from the corresponding author upon a reasonable request.
